# Integrating physiology into the first two years of a new osteopathic medical school curriculum

**DOI:** 10.3389/fphys.2023.1175662

**Published:** 2023-08-11

**Authors:** Natalie F. Slater, Irina Nizamutdinova, Blaine A. Jacobs, Robert T. Slater, Jessica M. Bradley

**Affiliations:** Clinical and Applied Biomedical Sciences Department, University of the Incarnate Word School of Osteopathic Medicine, San Antonio, TX, United States

**Keywords:** curriculum, education, medical, integration, undergraduate, physiology

## Abstract

The integrated curriculum is a hot topic in curriculum reform in undergraduate medical education; however, there are varying definitions of the term, and resources guiding the integration of specific disciplines throughout the first 2 years of undergraduate medical education in a learner-centered curriculum are limited. Our first class matriculated into our osteopathic medical school in 2017, and since then we have developed and implemented a learner-centered, integrated curriculum that begins on day one for our learners. This paper will discuss our experience with the development and implementation of the University of the Incarnate Word School of Osteopathic Medicine (UIWSOM) curriculum with specific emphasis on how we incorporate physiology into it.

## 1 Introduction

The refinement of medical education to benefit today’s learners requires teamwork. Whether this is the modification of a traditional curriculum or the development of a brand-new curriculum, faculty need to collaborate to design and implement a curriculum that engages the learner, helps the learner to develop their problem-solving and clinical reasoning skills, and assess the learner in a way that ensures competency. One major focus from the Carnegie Foundation for the Advancement of Teaching on curriculum reform, is that the curriculum should create an “opportunity for integrative and collaborative learning” (Irby et al., 2010). Since this report has been published, there is a lot of buzz around term “integrated curriculum”, but there still is varying information available on what this means or how to develop such a curriculum. Most of the current literature focuses on the integration of specific topics, such as ethics (Brunger and Duke, 2012), clinical skills (Brunger and Duke, 2012; Kondrashova et al., 2015), or lifestyle medicine (Phillips et al., 2015), into already developed curricula or courses. While this may be beneficial for established medical institutions, this does not provide guidance to those newer allopathic and osteopathic medical schools that are starting from scratch. Since the first class matriculated into the Doctor of Osteopathic Medicine (DO) program at our osteopathic medical school in 2017, we have been able to design and implement our curriculum from the ground-up.

In this paper, we will outline the learner-centered, integrated curricular structure at the University of the Incarnate Word School of Osteopathic Medicine (UIWSOM), describe the major philosophies underpinning our curriculum, explain how physiology is integrated with clinical and other basic sciences throughout the first 2 years, and discuss our experience with this endeavor, including challenges faced and future goals.

## 2 UIWSOM curricular structure and philosophy

### 2.1 UIWSOM curricular structure

The UIWSOM curriculum is divided into two phases. Phase I, years one and two, builds the foundation for applied biomedical sciences, clinical sciences, osteopathic principles and practice, and professional identity. Phase II, years three and four, further builds on clinical experiences through core, selective, and elective rotations.

The organization of the units in Phase I is based upon the systems shown in Table 1.

**TABLE 1:** Phase I units.

Year	Unit	Title
1	1	Foundation, Integration, and Transformation
	2	Musculoskeletal System, Touch, and Personhood
	3	Molecules, Cells, and Compassion
	4	Host Defense and Communication
	5	Gastrointestinal System, Nutrition, and Appetite
2	6	Circulation, Respiration, and Regulation
	7	Endocrine, Reproduction, and Respect
	8	Mind, Brain, and Behavior

We do not have courses for the individual disciplines; instead, we have the following curricular components:

• **Developing Osteopathic Clinical Skills (DOCS)** is designed to develop the skills necessary to practice osteopathic medicine, such as medical interviewing, physical and mental examination, documentation, communication, clinical reasoning, osteopathic principles and practice including osteopathic manipulative medicine, and clinical simulation.
• **Small Interactive Groups Sessions (SIGS)** consist of small group, learner-led case-based learning where learners collectively extract and compose objectives from cases, independently research the objectives, and then report their findings in a collaborative setting that strives for critical thinking.
• **Structures (STRX)** is designed to develop an understanding of the anatomical structure and function of the human body through focused dissection, prosected specimens, small group discussions, and imaging and histopathology.
• **Large Group Sessions (LGS)** are sessions with the entire class that are co-facilitated by faculty content experts and utilize higher-order learning activities to enable the integration of basic sciences, clinical sciences, and/or humanities.

### 2.2 UIWSOM curricular philosophy

From its inception, our curriculum was structured to align with the themes delineated in the Carnegie Foundation’s call for reform as well as adult learning theory. In order to explain how physiology is incorporated into the overall curriculum, to the LGS curricular components, and to the individual sessions in the first 2-years at UIWSOM, we will first elaborate on two central philosophies, integration and adult learning, that are rooted in our institutional culture and in our curricular design and delivery.

#### 2.2.1 Curricular integration

The models of integration that were used to initially develop our curriculum were that of horizontal and vertical integration. Horizontal integration is “integration across disciplines but within a finite period of time” (Brauer and Ferguson, 2015). The first 2 years of the UIWSOM curriculum achieves horizontal integration by combining pre-clinical basic sciences into interdisciplinary systems-based units. Horizontal integration is further accomplished by combining these basic sciences into individual sessions and learning activities. Vertical integration is “integration across time, attempting to improve education by disrupting the traditional barrier between the basic and clinical sciences” (Brauer and Ferguson, 2015). To achieve vertical integration, the first 2 years of the UIWSOM curriculum incorporates clinical sciences into the interdisciplinary systems-based units, into all curricular components, and into individual sessions. The UIWSOM curriculum has both horizontal and vertical integration and relies on building on and re-visiting concepts; however, true spiral integration requires that “foundational and clinical sciences interact equally throughout all phases of a curriculum” (Brauer and Ferguson, 2015). Although this is true for the Phase I curriculum, the Phase II curriculum more closely resembles the Z-shaped curriculum model (Wijnen-Meijer et al., 2009), which may be a direct result of the timeline of our school’s development since the Phase II curriculum is still undergoing significant expansion and refinement.

Though the level of integration may slightly vary in different units, curriculum components, and sessions, the majority of the Phase I curriculum lies at the multidisciplinary step of the integration ladder (Harden, 2000). The structure of the early UIWSOM curriculum, consisting of systems-based units with weekly themes and integrated curriculum components, make multidisciplinary integration at minimum easily attainable. The design and delivery of individual learning sessions is what can and often does push UIWSOM toward the interdisciplinary integration step. Through effective case design and team-teaching, there is a “further shift of emphasis to themes as a focus for the learning of and to the commonalities across disciplines as they relate to the theme” and “there may be no reference to individual disciplines” (Harden, 2000).

#### 2.2.2 Adult-learning

To promote the acquisition, retention, and application of knowledge, skills, and attitudes and ultimately the development of future physicians with improved critical thinking and clinical reasoning abilities, our curriculum structure and session design across all components of our curriculum are supported by adult learning theory (Taylor and Hamdy, 2013). The aim is to get learners to move past surface learning to a deeper understanding and to be able to put the content into practice. To that end, sessions across our curriculum involve experiential and self-directed learning with ample opportunity for application, self-assessment, feedback, and reflection.

We approach learning as an iterative cycle with clear and consistent facilitator and learner roles. Faculty provide scaffolding by way of learning outcomes (LO) and session objectives (SO) and pre-reading, followed by creation of sessions that require active learner participation, and development of integrated formative and summative assessments. Active learning strategies employed during sessions include but are not limited to concept mapping, think-pair-share, clinical case dissection or development, Socratic dialogue, and role-playing. With the foundation that “learning and thinking are social activities” and “discussion between individuals will increase the amount of practical knowledge”, small group activities are frequently utilized across all components of the curriculum, including within LGS (Taylor and Hamdy, 2013). Our physical resources, such as classrooms, were designed with this curricular structure in mind and are set up to support this style of delivery. For example, the interactive learning studio classroom where LGS occur accommodates the class and is arranged as 18 tables of eight to ten learners each. Every table has access to their own television display, white board, and microphone to enable participation.

## 3 Integration of physiology

### 3.1 Integrating physiology into the UIWSOM curriculum

**FIGURE 1:**
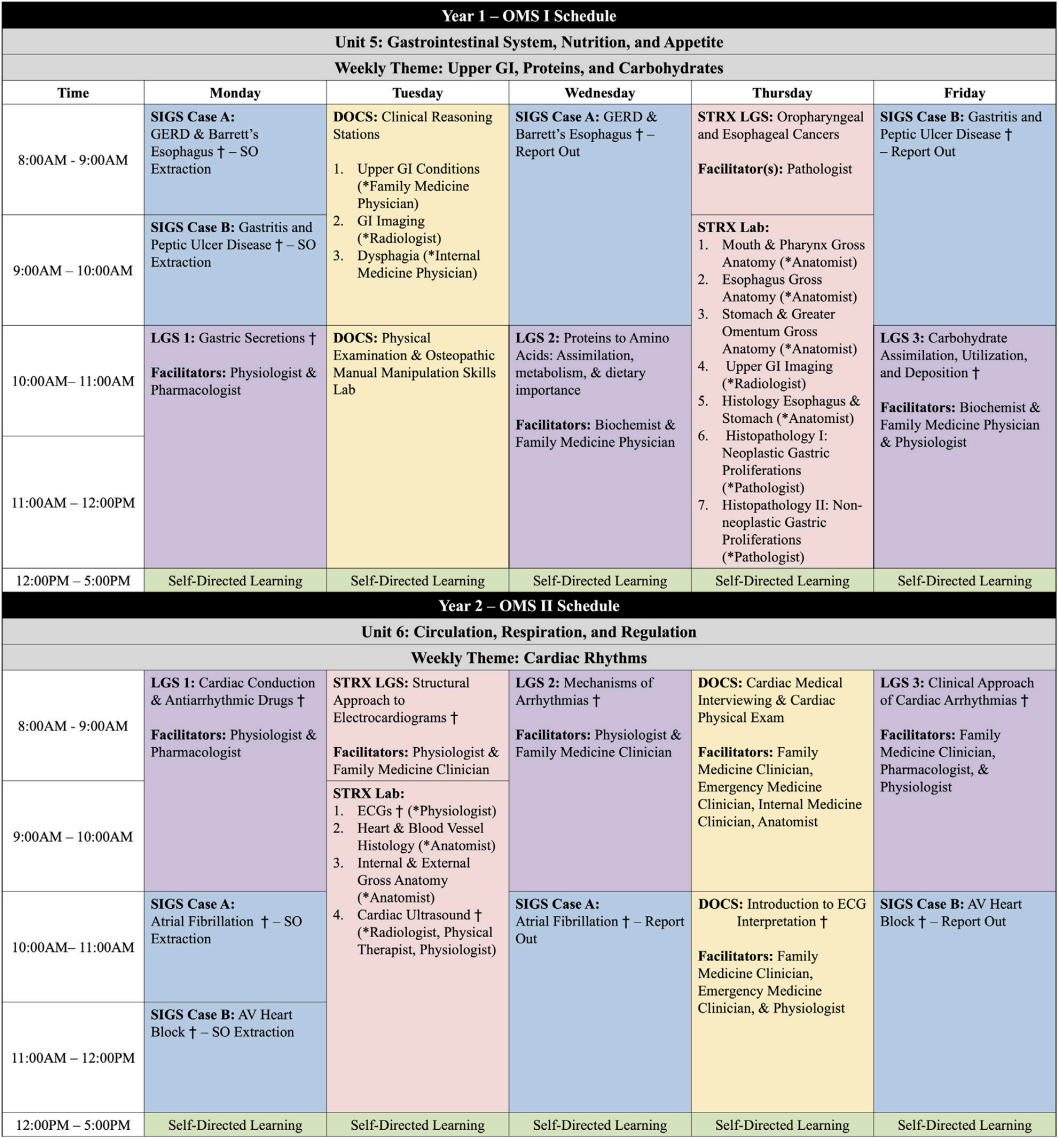
Weekly schedules for the Osteopathic Medical Student (OMS)-I and OMS-II learners in Phase I. Developing Osteopathic Clinical Skills (DOCS); Large Group Sessions (LGS); Small Interactive Group Sessions (SIGS); Structures (STRX) *Facilitators for Stations † Sessions where physiology content is included.

The initial development of the UIWSOM curriculum started 1 year prior to the first class matriculating and continues to occur year-by-year as our classes progress. The initial development involved a team of faculty and administration with a variety of backgrounds to outline the systems-based units. Then, multidisciplinary unit teams were created to identify and sequence the themes of the weeks within these units. The weekly themes were created to encompass the topics to be covered in each of the curricular components, and this multi-disciplinary approach encouraged horizontal and vertical integration early on in our development. A snapshot of a week in Unit 5 and Unit 6 is shown in Figure 1.

Because we do not have individual courses, a challenge with implementing individual disciplines into this style of curriculum is that faculty must cooperate and agree upon the content to include and to maintain the appropriate balance of basic science and clinical content. For us, this involved having all the physiologists first decide together on the core concepts and objectives that are needed for first- and second-year medical students and how they would ideally be arranged throughout the Phase I curriculum. Initially, we utilized the Medical Physiology Learning Objectives from the American Physiological Society as a primary guiding resource (Carroll et al., 2001); however, we tailored them to fit our needs and to allow integration with other disciplines. As we review and refine the curriculum, we often refer to the core competencies and concepts recognized by the American Association of Colleges of Osteopathic Medicine and the COMLEX-Level 1 United States blueprint to allow for continual alignment of our content with the national standards (AAMCOM, 2023; NBOME, 2023).

Once the core concepts for individual disciplines such as physiology were determined, in the multidisciplinary unit teams, we collectively organized these concepts to fit into the relevant weekly themes, the most appropriate curricular component, and to individual sessions. As the blueprint for each unit was formed, we then identified faculty content experts that would be responsible for developing and delivering individual sessions and for writing SIGS cases. To keep in alignment with our mission, professional identity formation, specifically physician personhood, was also integrated into each of the units in sessions or in SIGS cases by our physicians, medical humanists, ethicists, or medical jurisprudence faculty.

This entire process required compromise, flexibility, and openness from everyone involved. To assist while building out the basic and clinical sciences throughout Phase I, we set best practices for faculty, which we still abide by. First and foremost, the content in sessions is expected to be clinically relevant with major emphasis on the basic sciences. The basic science content supports current clinical applications, includes high-yield concepts, and is based upon nationally recognized standards for medical students. Clinical content is tailored to the level of a first- or second-year medical student, models the standard of care in medical practice, and correlates with the basic sciences. Finally, discussion of extremely rare diseases or outdated experimental basic or clinical sciences is typically limited.

### 3.2 Integrating physiology into LGS

Customarily, LGS are co-facilitated with two to three content experts from various disciplines and/or specialties. With significant planning, co-facilitators ensure that integration occurs throughout the entire session, rather than being two to three individual mini sessions. Faculty have academic freedom to choose how to best integrate and deliver the content and will often de-brief with co-facilitators after sessions to determine how to improve for the next academic year. Unless a major change is necessary and undergoes our curriculum revision process, the learning outcomes for each session typically remain consistent from academic year to year, to ensure standardization in curriculum mapping.

Our physiologists primarily co-facilitate sessions with pharmacologists, biochemists, and/or clinicians. When co-facilitated with another basic scientist, the session typically illustrates the relationship between the two disciplines. Similar to overall curriculum and unit development, a hurdle we often face in session development is how much or little of each discipline should be incorporated. Therefore, having clear and stable LO that support the topic of the session and theme of the week helps overcome this because co-facilitators know the overarching goals of the LGS and can adjust their content accordingly. For example, for the Gastric Secretions LGS that is held in Year 1 (Figure 1), we integrate physiology, pharmacology, and pathophysiology. This particular LGS utilizes the following high order LO based on Bloom’s taxonomy to complete the phrase “*At the end of the session, a learner is expected to be able to*”:

• Discuss the cellular and physiological mechanisms that stimulate/suppress gastric secretory products at the various stages of digestion.
• Examine how pKa and pH affect drug solubility and absorption.
• Characterize the pathophysiological mechanism for those diseases or conditions that alter acid production and damage the mucosa.
• Justify the pharmacological management of acid-peptic diseases.

To help in learner preparation and to provide learners background knowledge needed for the session, we give lower order, more granular SO that complete the phrase *“Prior to attending a session, a learner is expected to be able to.”* The SO align with the LO and the assigned pre-readings. Scheduled class time is typically limited to 4 hours per day to allow for adequate self-directed learning and session preparation by learners. LOSO documents for all curricular components except for SIGS are released two-weeks prior to the session. For each one-hour session in most components of the curriculum, we limit the number of LO to two, SO to six, and the number of pages of reading to 25 (Klatt and Klatt, 2011). The self-directed preparation should take learners approximately one to 2 hours per one-hour of class time. To abide by the pre-reading page limitations, we often assign sections of chapters rather than an entire chapter and pre-readings from multiple sources, which helps maintain integration of the session. For the Gastric Secretions LGS, we used pre-readings from a gastrointestinal physiology textbook, pharmacology textbook, and pathology textbook.

For the session itself, we create activities centered around the LO that require application of the disciplines and active learner participation. For example, in order to apply physiology, pharmacology, and pathophysiology in the Gastric Secretions LGS, learners within their small table groups build a clinical case, including clinical presentation, physical examination findings, and risk factors, on acid dysregulation. Learners present these cases to the larger class and solicit differential diagnosis or potential etiologies from their colleagues. Furthermore, during this LGS, learners’ diagram physiologic mechanisms of acid production and potentiation using white boards. Group discussion with Socratic questioning ensues relating these physiological functions back to the underlying pathophysiology depicted in their self-created cases. During this discussion, we then provide learners with a scenario to have them work though how gastric acid with and without an antacid can affect drug solubility and absorption. Continuing to use their white board drawing of the physiologic mechanisms as a foundation, several additional activities are then done to integrate the pharmacology of antacids, proton pump inhibitors, and histamine receptor blockers. These include large and small group discussions of how the mechanisms of action of these therapies are related to physiological function, how a fast or fed state can alter the efficacy of these therapies, and how these therapies could be used in the management of the conditions in their designed cases.

Through effective curriculum planning, we have been able to incorporate physiology content more broadly across an entire unit or week, which allows for spiraling and constructing more complex topics. For example, the Year 2 weeks on *Cardiac Rhythms* (Figure 1) has three LGS containing physiology. The first LGS of the week, *Cardiac Action Potentials and Antiarrhythmic Drugs,* sets the stage and uses physiological and pharmacological application to discuss how cardiac action potentials and conduction can be altered. The second LGS of the week, *Mechanisms of Arrhythmias*, builds on the physiology that was discussed in the first session, and learners correlate how alterations in impulse formation and impulse conduction can lead to various arrhythmias. In this session, we utilize electrocardiogram (ECG) images to have learners identify abnormalities on the ECG and to explain the underlying mechanisms for the arrhythmias. This LGS is not necessarily about identifying the arrhythmia but learning why and how the abnormality for the ECG resulted, based on the physiological mechanisms of cardiac conduction. In the third LGS of the week, *Clinical Approach of Arrhythmia*, we continue building on the physiological mechanisms of cardiac conduction through the use of clinical cases. While this session is designed specifically for diagnosis and management, integration back to physiology helps the learner further connect (1) the alterations in conduction to the patient’s clinical presentation and laboratory and diagnostic findings and (2) how the indicated antiarrhythmic drug alters the cardiac action potential to resolve the arrhythmia. During this week, the DOCS and STRX components of the curriculum incorporate physiology by performing and interpreting ECGs while relating these tasks to pertinent clinical and anatomic aspects.

Under the premises that “the success of integrated curriculum depends on implementation of integrated assessment” (Malik and Malik, 2011), we have developed weekly formative assessments and end-unit summative assessments utilizing clinical vignettes with free-response or short answer questions. Questions are mapped to SO, and learners are provided faculty-written suggested answers upon completion of the assessments for learner self-assessment. This style of assessment matches our teaching strategies and has requires learners to demonstrate critical thinking and competency in the material. This style of assessment has also enables integration on the assessments and even within questions.

## 4 Discussion

A common theme among institutions that have converted to an integrated curriculum is that development and implementation is a complex process that requires significant faculty time and collaboration (Muller et al., 2008; Malik and Malik, 2011). The initial organization and implementation of our curriculum required long hours in overall curricular, unit, and session planning meetings as well as to write and review SIGS cases. Now further into our development, we still have annual unit planning meetings with all involved faculty and planning and debrief meetings with co-facilitators for individual sessions. From academic year to year, these meetings allow us to ensure that integration is consistent and that we are adhering to our curriculum blueprint. Moving up the integration ladder necessitates increasing collaboration between faculty, a central curriculum organizational structure, and agreement between departments (Harden, 2000). Therefore, reflecting the mission and vision of the school and the curricular philosophy, the organizational structure at UIWSOM enables creation of a collaborative environment and does not consist of discipline-specific departments but rather two offices. The Office of Clinical and Applied Science Education focuses on the Phase I curriculum and the Office of Clinical Affairs focuses on the Phase II curriculum.

A crucial component to onboarding new faculty and training current faculty about our curricular model is faculty development. Our institution has implemented a collaborative, individualized, focused, and formative faculty development program (Bustamante-Helfrich et al., 2022) with a portion of this program involving peer observation. Utilizing peer coaches, we provide constructive feedback for individual LGS which has enhanced facilitation in the classroom. In addition, curriculum leaders hold monthly faculty development sessions based on the needs of the faculty.

Because active learning is a cornerstone of our curriculum, ensuring learner engagement has been and will continue to be a large focus of faculty development initiatives and curricular improvement processes. Historically, attendance has been required in all components of the curriculum except for LGS. More recently, attendance in LGS was made mandatory to increase attendance uniformity across sessions. Anecdotally, attendance at, learner preparation for, and participation in LGS seemed to be largely related to a range of factors, including learners’ perception of the importance of the content whether for future clinical practice or more immediately for national board examinations, consistent presence of the material on end-of-unit examinations, learners’ ability to clearly understand the expectations and objectives of the session, and individual facilitators’ delivery methods. Motivating learners to engage is often ideally accomplished by providing faculty-resources and classroom activities that align with the content and format that appear on assessments; however, there are times when faculty struggle with sessions or activities not going as planned or where learners reach out to third-party learning materials for lower-order foundational knowledge in place of faculty-recommended resources and active session participation. Although utilizing free response and short answer questions has been beneficial in assessing learner competency and encouraging high order thinking, there have been challenges. The amount of content that can be tested is limited, the grading workload on the faculty is significant, and providing feedback to learners takes time. Dialogue surrounding the methods for holding learners accountable in our learner-centered curriculum, such as through various assessment strategies, and using external learning resources to supplement our curriculum is ongoing.

While we have executed and demonstrated apparent success in designing and implementing a new curriculum designed around integration, our future goals will be to determine whether the intended benefits of integration are actualized, to ensure sustainability, and to refine the curriculum as needed. Essential outcomes to further analyze are clinical performance, overall and discipline-specific achievement of competencies on internal and national assessments, and learner feedback. We will also need to maintain and frequently review an updated, detailed curriculum map containing learning outcomes, session objectives, sequence in curriculum, content delivery methods, and assessment methods as any necessary changes are made. Making this easily accessible to both faculty and learners will allow content experts to identify gaps in content, preserve the presence of basic sciences within the curriculum, and to adjust the timing and integration of sessions. It will also make the intentional integration and connections between content more readily apparent to learners (Brauer and Ferguson, 2015). Evaluating our assessment methods, exploring other published strategies and tools for assessment within integrated curricula, and providing additional opportunities for assessment within the curriculum will be future areas of focus. Lastly, concentrating efforts on better incorporating basic science such as physiology into the third and fourth years will move us closer to the ideal of the true comprehensive integrated curriculum and to addressing a common pitfall frequently encountered within the integrated curriculum (AAMC, 2001).

## Data Availability

The original contributions presented in the study are included in the article/Supplementary material, further inquiries can be directed to the corresponding author.
